# Estimating clinical transitions in patients with Alzheimer's using Instrumental Activities of Daily Living (IADL)

**DOI:** 10.1002/alz70857_107058

**Published:** 2025-12-25

**Authors:** Ying Wang, Joel Reisman, Brant Mittler, Dan Berlowitz, Peter J Morin, Raymond Zhang, Amir Abbas Tahami Monfared, Quanwu Zhang, Weiming Xia

**Affiliations:** ^1^ Wentworth Institute of Technology, Boston, MA, USA; ^2^ University of Massachusetts Lowell, Lowell, MA, USA; ^3^ Geriatric Research Education & Clinical Center, VA South Texas Healthcare System, San Antonio, TX, USA; ^4^ Boston University Chobanian & Avedisian School of Medicine, Boston, MA, USA; ^5^ Clinical Evidence Generation, Deep Human Biology Learning, Eisai Inc., Nutley, NJ, USA

## Abstract

**Background:**

Previously, we proposed disease staging using the Lawton‐Brody Instrumental Activities of Daily Living Scale (IADL) in patients with mild cognitive impairment (MCI) or Alzheimer's dementia (AD), using a crosswalk against the Mini‐Mental State Examination (MMSE). This study aimed to estimate disease trajectory based on proposed IADL Alzheimer's stage cut‐off scores.

**Method:**

Patients with MCI or AD who had an IADL administered between 2016–2024 were identified from the US Veteran's Affairs Healthcare System administrative database. IADL cut‐offs were derived in four ways: descriptive values, and least squares (LS) means from linear, categorical, and repeated measures analysis; this study used linear LS means, grouped as >5.4, 4.7‐5.4, 4.1‐<4.7, 2.7‐<4.1, <2.7 for normal, MCI, mild, moderate, and severe stages, respectively. IADL scores were analyzed using mixed effects statistical modeling with patient's state of residence (reflecting care location) as a random effect, controlling for demographics. Disease stage transition over time was estimated, allowing nonlinear trends and interactions with initial diagnosis of MCI vs AD.

**Result:**

Our sample (*N* = 90,114) had a mean age of 80.2 years (97.5% men, 14.1% Black, and 17.0% Hispanic). MCI patients (*n* = 54,569) had a mean age of 79.2 years (97.4% men, 14.5% Black, and 13.1% Hispanic); AD patients (*n* = 35,545) were aged 81.7 years (97.7% men, 13.4% Black, and 22.9% Hispanic). Acetylcholinesterase inhibitors and/or memantine was used in 28% of patients overall, and in 23.6% and 34.9% of patients with MCI and AD, respectively. In the MCI group, estimated transition times were 5.2 years from MCI to mild, 2.9 years from mild to moderate, and 4.8 years from moderate AD to severe stages, based on the IADL cut‐offs. In the AD group, estimated transition times were 3.5 years from mild to moderate, and 5.3 years form moderate to severe stages.

**Conclusion:**

Functional deterioration appeared to progress to severe stage within 9 years for AD and within 13 years for MCI. Polynomial growth curves may tend to skew transition times at more advanced stages; however, trajectory estimations provide an alternative measure of disease progression with IADL, a broadly used assessment in clinical practice, to assess functional decline corresponding to cognitive deterioration.

